# Whether and how will using social media induce social anxiety? The correlational and causal evidence from Chinese society

**DOI:** 10.3389/fpsyg.2023.1217415

**Published:** 2023-09-29

**Authors:** Feng Yang, Minyan Li, Yang Han

**Affiliations:** ^1^Department of Teacher Education, Taishan University, Tai’an, Shandong, China; ^2^Department of Psychology, Beijing Sport University, Beijing, China

**Keywords:** social media, social anxiety, social comparison, self-esteem, cyberpsychology

## Abstract

**Background:**

Prior literature has well established the relationship between social media use and social anxiety, but little attention has been paid to the underlying mechanisms. Additionally, the causal evidence concerning the effect of social media use on social anxiety is scarce.

**Objective:**

Given that, two studies were conducted to examine the effect of social media use on social anxiety and the underlying mechanisms.

**Methods and results:**

In Study 1, with 470 undergraduates as participants, we applied the questionnaire survey to investigate the relationship between social media use and social anxiety. The results showed that higher social media use intensity was significantly related to higher social anxiety, and social media use was related to social anxiety via two possible mediation paths: (1) social media use → upward social comparison → social anxiety, (2) and social media use → upward social comparison → self-esteem → social anxiety. In Study 2, with 180 undergraduates as participants, we conducted a lab experiment, in which participants were assigned to the experimental (exposed to the content that undergraduates frequently access on social media) or control (exposed to landscape documentaries) condition, and then measured their upward social comparison, self-esteem and social anxiety. The results showed that participants in the experimental condition reported higher social anxiety than those in the control condition, demonstrating the causality between social media exposure and social anxiety. The subsequent mediation analysis basically replicated the findings of Study 1. That is, upward social comparison played the mediating role between social media exposure and social anxiety, and upward social comparison and self-esteem played the chain-mediating role between them.

**Conclusion:**

The current research firstly demonstrated the causality between social media use and social anxiety in Chinese society, and also revealed the mediating mechanisms between them, which would deepen our understanding of how social media use will increase social anxiety.

## 1. Introduction

Imagine that you meet your best friend again after a long separation and you have a nice dinner together. Now, you are so excited and happy, and want to share this wonderful experience with others around you. What will you do? In China-Mainland, the answer may be sharing your reunion on WeChat Moments,^1^ which allows users to share their personal photos, wonderful life experiences, and feelings at the moment with online friends, and also allows users to view the content shared by other users. Across the world, there are numerous online-platforms having similar functions with WeChat, such as Facebook, Instagram, YouTube, MicroBlog and so on, and these platforms can be collectively called as social media (Kaplan and Haenlein, 2010; Carr et al., 2016). During the past two decades, social media has become a part of everyday life and yields a profound influence on our cognition, mood, and behaviors (Olson et al., 2012; O’Day and Heimberg, 2021). A large body of research suggests that social media function as a double-edged sword: on one hand, social media helps people free from the restriction of time and space, and communicate with each other more effectively; on the other hand, excessively using social media also brings some detrimental effects on people’s mental health (She et al., 2023).

Past research demonstrates that individuals with high social media use intensity tend to report high social anxiety (e.g., Davidson and Farquhar, 2014; Dobrean and Pasarelu, 2016; Jiang and Ngien, 2020). However, prior research concerning social media use and social anxiety in general adopts the questionnaire survey to provide correlational evidence for the correlation between them, resulting in the lack of causal evidence about the effect of social media use on social anxiety. In other words, the questionnaire survey method allows us to test the correlation between social media use intensity and social anxiety, but fails to make us confirm whether higher social media use intensity will lead to higher social anxiety.

Additionally, it is somewhat surprising that researchers so far have paid little attention to the underlying mechanisms between social media use and social anxiety (Jiang and Ngien, 2020). Thus, to make clear how social media use in daily life will induce users’ social anxiety, more work should be done to uncover the mechanisms behind the effect of social media use on social anxiety.

Finally, prior research with respect to social media use and social anxiety is exclusively conducted in Western society and the involved social media platforms include Facebook and Instagram, which both are popular social media platforms in Western society. However, due to the regulation of Chinese government, the mentioned above social media platforms failed to provide services in China-Mainland. In this situation, we actually do not know whether the results-pattern observed in Western society can be generalized to Chinese society. Moreover, the cultural differences about social anxiety have been found to exist between Eastern and Western societies (Heinrichs et al., 2006). Therefore, it is necessary for us to examine the possible effect of using social media on social anxiety in Chinese society.

To fill the above gaps existing in the field of social media use and social anxiety, we aimed to conduct two studies—a questionnaire survey and a lab experiment, to exclusively examine the effect of using social media on social anxiety in China-Mainland, thus firstly providing both correlational and causal evidence for the effect of social media use on social anxiety and the underlying mechanisms.

## 2. Literature review

### 2.1. Social media

According to the definition proposed by Kaplan and Haenlein (2010), social media can refer to any social networking sites or various of Internet-based applications, which was considered to developed based on the Web 2.0 platforms and the ideology of “User Generated Content” (Kaplan and Haenlein, 2010; Tartari, 2015). A typical characteristic of social media is that it allows all users to participate in the creation of the presented content and communicate with each other among social media users. Now, social media has become a part of people’s lives in modern society, through which people can maintain interpersonal relationships, document those meaningful moments in life, gain knowledge and information from the external world, and share whatever you are willing to share with others (Boyd and Ellison, 2007). Besides, it is readily for social media users to perceive social support from others on the platform, which is considered to be a protective factor for our self-esteem and subjective well-being (Rozzell et al., 2014; Carr et al., 2016; Tao and Cheng, 2018).

Despite the fact that people benefit a lot from social media, researchers also reveal some detrimental influences on individuals’ mental health and social adaptation resulting from excessive social media (O'Keeffe et al., 2011; Amedie, 2015; Tartari, 2015). For example, previous research consistently shows that individuals who spend too much time on Facebook are inclined to exhibit classic symptoms of depression, called “Facebook depression” (Jelenchick et al., 2013; Steers et al., 2014; Alfasi, 2019). Additionally, due to the positive self-presentation tendency on social media, individuals with excessive social media use often suffer from upward social comparison and mistakenly perceive that they are inferior than others round them, which further harms their self-esteem (Jan et al., 2017). In some cases, social media provides an ideal place for some criminals to hide their identity and spread criminal ideas, such as cyber bullying, cyber terrorism, and drug dealing (Amedie, 2015).

### 2.2. Social media and social anxiety

The term social anxiety refers to a phenomenon existing in social situations that one person has a strong desire to make a good impression on others while the person is quite not sure whether he/she has the ability to this end (Yen et al., 2012; Morrison and Heimberg, 2013). The cognitive-behavioral model of social anxiety posits that individuals with social anxiety tend to exhibit three tendencies: (1) applying an excessively high standard for their performances in social situations, (2) presupposing that the encountered others will give negative evaluations for their social performances, and (3) being inclined to believe that others’ evaluations about themselves are true (Clark and Wells, 1995; Rapee and Heimberg, 1997). As a result, when interacting with others in social situations, socially anxious individuals often experience fear or anxiety that they will be negatively evaluated or judged by others (Clark and Wells, 1995; Alden and Taylor, 2004; Hofmann, 2007; O’Day and Heimberg, 2021). And for these individuals, this excessive fear for poor social performances will further reduce the possibility to develop new interpersonal-relationships and also have a detrimental influence on the existing interpersonal-relationships (O’Day and Heimberg, 2021). It should be pointed out, despite the fact that social anxiety is detrimental to developing normal social interaction, anxiety symptoms have been widely observed in nonclinical samples and most people actually have experienced more or less social anxiety in daily life (Purdon et al., 2001).

Prior literature has documented that there is a significant correlation between social media use and social anxiety (Davidson and Farquhar, 2014; Dobrean and Pasarelu, 2016; Jiang and Ngien, 2020; Sternberg et al., 2020; O’Day and Heimberg, 2021). For instance, in a questionnaire survey conducted in Singapore, researchers found that those participants with higher Instagram use intensity were inclined to report higher social anxiety, suggesting excessive social media use may elevate users’ social anxiety (Jiang and Ngien, 2020). Similarly, in another study by Davidson and Farquhar (2014), researchers examined the relationship between Facebook use intensity and social anxiety and revealed a significant correlation between them. Even for those individuals having suffered from social anxiety, using social media will further enhance their social anxiety although they initially intend to compensate for their “deficits” in social skills via the online interactions on social media (Carruthers et al., 2019). It should be pointed out, several existing studies examining the effect of social media use on social anxiety adopted a single questionnaire approach, so such studies in nature are a correlational design (Davidson and Farquhar, 2014; Qiu et al., 2017; Jiang and Ngien, 2020). And a single study conducted in the lab consisted of clinical samples (individuals suffering from social anxiety) but not community samples (Carruthers et al., 2019). As a consequence, we so far cannot draw a causal inference about whether social media use will increase social anxiety of users in daily life. To fill this gap, the current research sought to provide first evidence for the causality between social media use and social anxiety. Correspondingly, our first hypothesis was that,

*Higher social media use intensity would lead to higher social anxiety (Hypothesis 1)*.

### 2.3. Social media, upward social comparison, and social anxiety

According to the propositions of social comparison theory, we often intentionally or unconsciously compare ourselves with others around us in order to assess our self-worth or gain our self-enhancement (Festinger, 1954). When comparing ourselves with someone who is better than us on specific domains, we can call this comparison as upward social comparison; in contrast, when comparing ourselves with someone who is worse than us on specific domains, we can call this comparison as downward social comparison (Wills, 1981; Wood, 1989). Past research suggests that compared to downward social comparison, upward social comparison is more likely to be associated with a series of negative health outcomes, such as envy, lower well-being, depressive symptoms, and lower self-esteem (Feinstein et al., 2013; Charoensukmongkol, 2018; Tao and Cheng, 2018; Alfasi, 2019; Schmuck et al., 2019). In contrast, comparing oneself with others inferior than the self has been found to be helpful for elevating self-esteem, receiving mental gratifications, and maintaining good mood states (Gibbons and Gerrard, 1989; Lew et al., 2007; Huang and Zhou, 2018). Notably, although a large body of research demonstrates the negative effect of upward social comparison and the positive effect of downward social comparison, the opposite patterns are also possible in given circumstances. As an example, for an individual with a self-improvement motive, comparing the self with a better one will provide hope and inspiration for the individual (Wood, 1989).

On social media, users can autonomously determine which aspects of them will be presented on the platform, and if necessary, they also can employ the beauty filter to construct perfect self-images following their aesthetic preference (Arroyo and Brunner, 2016; Yao et al., 2020). As a result of this characteristic of social media, it is a common phenomenon that social media users tend to present positive self-presentations on various of social media platforms (Haferkamp and Krämer, 2011). In this situation, social media users often mistakenly perceive that the lives of others always seem to be exciting and colorful, but their own lives seem to be mediocre and boring, which will result in the generation of upward social comparison (Chou and Edge, 2012; Steers et al., 2014).

Upward social comparison, a phenomenon commonly observed on social media platforms, has been found to be a risk factor inducing social anxiety (for a review, see McCarthy and Morina, 2020). The Cognitive-behavioral model of social anxiety has defined the fear of evaluation as a core feature of the disorder, and upward social comparison seems to readily induce this fear, especially the fear of negative evaluations from others (Clark and Wells, 1995; Rapee and Heimberg, 1997; Weeks et al., 2009; McCarthy and Morina, 2020). For example, Antony et al. (2005) used a questionnaire survey method to examine the relationship between social comparison and social anxiety among individuals with high and low social anxiety, and found that for individuals in both groups, upward social comparison was significantly and positively correlated with social anxiety. In another lab experiment by Mitchell and Schmidt (2014), researchers asked participants to view a profile of a fellow student with excellent performance (the upward social comparison condition) or with normal performance, and then measured participants’ social anxiety via the Social Interaction Anxiety Scale (Mattick and Clarke, 1998). The results showed that male participants with social anxiety tended to report higher social anxiety after experiencing upward social comparison in the lab. Following the above reasoning, we proposed our second hypothesis that,

*Using social media may have an influence on social anxiety via the mediating role of upward social comparison (Hypothesis 2)*.

### 2.4. Social media, upward social comparison, self-esteem, and social anxiety

In addition to directly inducing social anxiety, upward social comparison may also lead to lower self-esteem of social media users, and lower self-esteem may further lead to higher social anxiety. Krause et al. (2021) have proposed that social comparison can produce the opposite influences on self-esteem depending on the comparison direction—upward social comparison will harm self-esteem and downward social comparison will benefit self-esteem. Consistent with this proposition, empirical research revealed that, due to the positive self-presentation on social media, social media users in general experienced upward social comparison rather than downward social comparison when they use social media (Jan et al., 2017). And upward social comparison has been widely demonstrated to be correlated with lower self-esteem (Mitchell and Schmidt, 2014; Alfasi, 2019; Midgley et al., 2021). As an example, using an experimental approach, researchers found that participants reported lower self-esteem when they were assigned to the upward comparison condition (reading an introduction about an excellent college student) than the downward comparison condition (reading an introduction about an average college student; Mitchell and Schmidt, 2014).

Sociometer theory proposes that self-esteem represents “an internal, subjective gauge of interpersonal acceptance and Rejection”—high self-esteem means an individual perceives good interpersonal relationships and the individual can receive social acceptance from others, while low self-esteem means an individual perceives poor relationships and the individual may suffer from social exclusion (Leary and Baumeister, 2000; Leary, 2005). So, according to this theory, due to the threat of social exclusion, low self-esteem should have a close connection with social anxiety. In line with the proposition of the sociometer theory, empirical research also suggests that individuals with lower self-esteem tend to report higher social anxiety (van Tuijl et al., 2014; Fatima et al., 2017). Recently, with 388 Singaporeans as participants, Jiang and Ngien (2020) used the questionnaire survey to clarify the mechanisms underlying the relationship between Instagram use intensity and social anxiety, and found that upward social comparison on social media had a significantly negative prediction on self-esteem and self-esteem further had a significantly negative prediction on social anxiety. On the basis of previous research, we hypothesized that,

*Using social media may yield an influence on users’ social anxiety via the chain-mediating role of upward social comparison and self-esteem (Hypothesis 3).*

## 3. The current research

As we have documented in the beginning section, although there has been a large body of research demonstrating the significant correlation between social media use and social anxiety, the causal evidence between them is still scarce, which makes us fail to provide causal evidence for the effect of social media use on social anxiety. Additionally, there is little research exploring the acting mechanisms between social media use and social anxiety (Jiang and Ngien, 2020). Given the above considerations, we conducted two studies to examine the effect of social media use on social anxiety and the mechanisms underlying the effect. In Study 1, we employed the questionnaire survey to examine the correlation between social media use and social anxiety, and the mediating roles of upward social comparison and self-esteem between them, thus providing preliminary evidence for our hypotheses. In Study 2, we conducted a lab experiment to examine the causal relationship between social media use and social anxiety, and the mediating roles of upward social comparison and self-esteem, thus providing compelling evidence for our hypotheses.

## 4. Study 1

To provide initial evidence for our hypotheses, in Study 1, we conducted a questionnaires survey in Study 1 to examine the relationship between social media use intensity and social anxiety, and the mediating roles of upward social comparison and self-esteem between them.

### 4.1. Participants

Participants of Study 1 were 470 college students (182 males, 282 females, 6 participants did not provide gender information) from a university in Shandong Province of China-Mainland, whose average age was 20.99 years (*SD* = 1.33), ranging from 18.58 to 30.08 years. All participants took part in this survey on the voluntary basis and they signed the informed consent prior to the formal survey. Participants could receive 5 RMB as a return (approximately 0.8 USD) after completing the survey. Eight participants (3 males, 5 females) were regarded as invalid participants resulting from their excessive answer omissions, so there were 462 participants involved in final data analysis.

### 4.2. Measures

#### 4.2.1. The measure of social media use intensity

In the current research, the modified Facebook use intensity scale was used to assess participants’ social media use intensity. Specifically, to measure individuals’ use intensity of social media in Chinese society, Qiu et al. (2017) modified the Facebook use intensity scale developed by Ellison et al. (2007) to generate the social media use intensity scale. In the modified scale, the item “about how many total Facebook friends do you have at MSU or elsewhere” existing in the original scale was deleted, and the term “Facebook” was entirely replaced by the term “social media”. As a result, the social media use intensity scale in the current research includes six items—one item assesses the average time spent on social media per day and the other five items assess individuals’ attitudes toward social media use (e.g., *social media has become part of my daily routine*). For each item, participants needed to indicate their agreement on the 7-point scale (1 = *strongly disagree*, 7 = *strongly agree*). The social media use intensity of participants was assessed by summing the scores on all items. In the current research, the internal consistency coefficient of the scale was 0.72.

#### 4.2.2. The measure of upward social comparison

The Chinese version of the Iowa-Netherlands Comparison Orientation Measure was used to assess the tendency of upward social comparison, which was developed by Bai et al. (2013) on the basis of the original scale by Gibbons and Buunk (1999). In line with previous research (Qiu et al., 2017; Kong et al., 2021), only the ability subscale was used in the current research and a total of 6 items were included in the subscale [e.g., *I often compare how I am doing socially (e.g., social skills, popularity) with other people*]. For each item, participants needed to report their agreement on the 5-point scale (1 = *strongly disagree*, 5 = *strongly agree*) with higher scores indicating higher social comparison tendency (the item 5 was reversely scored). The internal consistency of the scale was 0.78.

#### 4.2.3. The measure of self-esteem

The Chinese version of the Rosenberg Self-Esteem Scale was employed to measure participants’ self-esteem (Rosenberg, 1965; Ji and Yu, 1999). The scale includes 10 items and participants needed to indicate their agreement for each item on the 4-point scale (1 = *strongly disagree,* 4 = *strongly agree*). It should be pointed out that, when participants filled out the scale, we instructed them to report their evaluations about themselves after using social media rather than their evaluations about themselves in general. After the item 3, 5, 8, 9, and 10 were reversely scored, self-esteem score was calculated by summing the score on each item, with higher scores indicating higher self-esteem. The Cronbach’s α of the scale was 0.83.

#### 4.2.4. The measure of social anxiety

The Chinese version of the Interaction Anxiousness Scale developed by Leary (1983) was used to measure the social anxiety of participants (Leary, 1983; Peng et al., 2004). The scale contains 15 items (e.g., *I get nervous when I must talk to a teacher or boss*) and participants needed to indicate their agreement on the 5-point scale (1 = *strongly disagree*, 5 = *strongly agree*). After the item 3, 6, 10, and 15 were reversely scored, we assessed participants’ social anxiety by summing the score on each item, with higher scores indicating more social anxiety. In the current research, the internal consistency coefficient of the scale was 0.87.

#### 4.2.5. The measure of demographic information

In addition to the measures of the above key variables, we also measured participants’ demographic information, including their age, gender, residential location (city/country), and subjective social status. One thing we wanted to explain was that, although parental income, job, and education were commonly used to represent social status of a family (e.g., Pinderhughes et al., 2000; Suleman et al., 2012), considering that most college students in China-Mainland actually are not clear how much their family earns each year, we did not assess participants’ objective social status in the current research. Instead, we asked participants to report their subjective social status by marking an “X” next to one of 10 rungs on a ladder to indicate their own social class rank relative to other college students all over China (Kraus et al., 2009).

### 4.3. Procedure

We carried out our questionnaire survey in a group of 40~60 participants. Before the formal survey, we told participants that we were conducting a survey about social media use and social anxiety. We also explicitly alleged that the collected data was used only for academic purpose, and if participants were willing to continue the survey, they needed to assign the informed consent. After that, we instructed participants to fill out several scales mentioned above and also report their demographic information. When they completed all measurements, they would receive their rewards (5 RMB). The whole survey lasted for approximate 10–15 min.

### 4.4. Results

#### 4.4.1. Descriptive results

We applied the SPSS 23.0 to sort the database and generate descriptive results. The correlations among variables were presented in Table 1. As shown in Table 1, social media use intensity was significantly and positively correlated with upward social comparison and social anxiety, *r*s = 0.23, 0.20, respectively, *p*s < 0.01. Upward social comparison was found to be significantly and negatively correlated with self-esteem, *r* = −0.22, *p* < 0.01, but positively correlated with social anxiety, *r* = 0.25, *p* < 0.01. In addition, self-esteem was significantly and negatively correlated with social anxiety, *r* = −0.37, *p* < 0.01.

**Table 1:** The correlations among variables in Study 1.

	Social media use intensity	Upward social comparison	Self-esteem	Social anxiety	Subjective social status	Age
Social media use intensity	1					
Upward social comparison	0.23^**^	1				
Self-esteem	−0.09	−0.22^**^	1			
Social anxiety	0.20^**^	0.25^**^	−0.37^**^	1		
Subjective social status	−0.04	0.07	0.34^**^	−0.17^**^	1	
Age	0.06	0.04	−0.18^**^	0.14^**^	−0.16^*^	1

^*^p < 0.05, ^**^p < 0.01.

In addition to the above significant correlations, an independent-samples *t-*test showed that female participants reported higher social anxiety than male participants (*M*_female_ = 49.94, *M*_male_ = 45.19), *t*(460) = 5.67, *p* < 0.001, *d* = 0.54, displaying the same pattern with previous research (Asher et al., 2017). Another independent-samples *t-*test showed that male participants tended to report higher subjective social status than female participants, *t*(460) = 3.37, *p* = 0.001, *d* = 0.32. Additionally, we found there was a significantly negative correlation between age and subjective social status, *r* = −0.16, *p* < 0.05, suggesting that senior college students perceived lower subjective social status than younger college students.

#### 4.4.2. The mediating roles of upward social comparison and self-esteem

The Macro PROCESS (model 6) by Hayes (2013) was applied to examine the possibly mediating effects of upward social comparison and self-esteem between social media use intensity and social anxiety. The process tests the mediating effect by using the bootstrapping method (5,000 bootstrapped resampling in this study) to create a 95% confidence interval, and if the interval does not contain a zero, the mediating effect will be considered to be reliable (Preacher and Hayes, 2008). Prior to the mediation analysis, we conducted the multicollinearity test and found that tolerance values and variance inflation factors were greater than 0.20 (0.79–0.98) and less than 10 (1.07–1.25), respectively. So, there were no problems with respect to multicollinearity between variables (Kutner et al., 2004). Additionally, the possible effects of demographic variables (gender, age, and subjective social status) were controlled when performing the mediating effect analysis.

**Figure 1:**
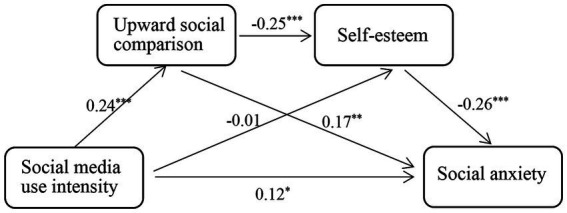
The mediating mechanisms between social media use intensity and social anxiety in Study 1 were illustrated schematically. ^*^p < 0.05, ^**^p < 0.01, ^***^p < 0.001.

The results showed that the overall model was significant, *R*^2^ = 0.07, *F*(5, 456) = 6.52, *p* < 0.001. As shown in Table 2, the direct effect between social media use intensity and social anxiety was significant, *f* = 0.13, 95% CI [0.04, 0.21]. Additionally, the indirect effect between social media use intensity and social anxiety via the mediating role of upward social comparison was significant, *f* = 0.04, 95% CI [0.02, 0.07]. As illustrated in Figure 1, social media use intensity significantly and positively predicted upward social comparison, *β* = 0.24, *p* < 0.001, and upward social comparison further significantly and positively predicted social anxiety, *β* = 0.17, *p* < 0.01. In addition to the mediating role of upward social comparison, we also found the chain-mediating role of upward social comparison and self-esteem between social media use intensity and social anxiety, *f* = 0.02, 95% CI [0.01, 0.03]. As displayed in Figure 1, social media use intensity significantly and positively predicted upward social comparison, *β* = 0.24, *p* < 0.001, and upward social comparison significantly and negatively predicted self-esteem, *β* = −0.25, *p* < 0.001. The self-esteem, in turn, further significantly and negatively predicted social anxiety, *β* = −0.26, *p* < 0.001.

**Table 2:** Direct and indirect effects between social media use intensity and social anxiety in Study 1.

	Effect size	Boot SE	95%LLCI	95%ULCI	Ratio
Direct effect					
Social media use intensity → social anxiety	0.13	0.04	0.04	0.21	67.36%
Indirect effect					
Social media use intensity → upward social comparison → social anxiety	0.04	0.01	0.02	0.07	20.73%
Social media use intensity→ upward social comparison → self-esteem → social anxiety	0.02	0.005	0.01	0.03	10.36%
Social media use intensity→ self-esteem → social anxiety	0.003	0.01	−0.02	0.03	1.55%

SE, standard error; LLCI, lower limit of confidence interval; ULCI, upper limit of confidence interval.

### 4.5. Discussion

In Study 1, we used a questionnaire survey to examine the relationship between social media use and social anxiety, and the underlying mediating-mechanisms. Consistent with Hypothesis 1, we found that there was a significantly positive correlation between social media use and social anxiety, which implies that individuals with high use intensity of social media were more likely to display social anxiety than individuals with low use intensity of social media.

Besides the direct effect between social media use intensity and social anxiety, we also found that higher social media use intensity was significantly correlated with higher upward social comparison, showing the same results-pattern with previous research (Lee, 2014; Jan et al., 2017). The higher upward social comparison, on one hand, directly induced social media users’ social anxiety, resulting in the mediating role of upward social comparison between social media use and social anxiety (Hypothesis 2); on the other hand, the higher upward social comparison was further correlated with lower self-esteem and lower self-esteem finally induced social anxiety, thus supporting the chain-mediating role of upward social comparison and self-esteem between social media use and social anxiety (Hypothesis 3).

It should be pointed out, Study 1 employed the questionnaire survey to examine the relationship and the underlying mechanisms between social media use and social anxiety, which should belong to the correlational research-design. As we have discussed in the introduction section, the correlational research-design allowed us to test whether there was a significant correlation between social media use and social anxiety, but did not enable us to confirm the causality between them. So, Study 1 indeed provided preliminary supports for our hypotheses. Given that, we aimed to conduct a lab experiment to further demonstrate the causality between social media use and social anxiety.

## 5. Study 2

To provide more convincing evidence for our hypotheses, we conducted a lab experiment—Study 2, in which we randomly assigned participants to the experimental or control condition and asked them to watch approximate 15 min videos shared on Bilibili,^2^ a social media platform widely popular among Chinese college students. In social media exposure condition (the experimental condition), the 15 min videos consisted of three different vlogs: one concerning fitness experiences-sharing, one concerning postgraduate entrance examination experiences-sharing, and the last one concerning travel experiences-sharing. In the control condition, the 15 min videos consisted of three vlogs about natural scenery: one called *Overlooking China*, one called *Beautiful China*, and the last one called *Wild China*. After that, we measured participants’ upward social comparison, self-esteem, and social anxiety. We provided more detailed introductions in the following *materials* and *procedure* sections.

### 5.1. Participants and design

We determined the sample size of Study 2 based on the calculation of G*power 3.1 (Faul et al., 2009), which suggested that the presupposed medium effect size *d* = 0.8 and the significance at 0.05 level required at least 88 participants in each condition to conduct an independent-samples *t-*test. Given that, we finally recruited 180 college students (79 males, 101 females, *M*_age_ = 19.74, *SD* = 0.93, ranging from 18.33 to 22.42) to participate in Study 2. As a return for their participation, they would receive 10 RMB (approximate 1.4 USD) when they complete all tasks. Due to excessive answer omission, 5 participants were regarded as invalid participants and excluded from the following data analysis. As a result, a total of 175 participants (79 males, 96 females) were included in final data analysis.

### 5.2. Materials

#### 5.2.1. The manipulation of social media exposure

Inspired by Tiggemann and Zaccardo (2015), in Study 2, we manipulated social media exposure of participants by inviting them to watch different short videos shared on Bilibili depending on the assigned conditions. In the experimental condition, the three videos presented for participants included fitness experiences-sharing, postgraduate entrance examination experiences-sharing, and travel experiences-sharing. Prior literature has documented that sharing fitness and travel experiences on social media is popular among young people (Tiggemann and Zaccardo, 2015). This is why the fitness experiences-sharing and travel experiences-sharing were included in the experimental condition. Additionally, considering that the participants of Study 2 were college students, the study also included the postgraduate entrance examination experiences-sharing. According to a survey from Beijing News (2019), it is common for college students to share and view some tips about important exams (e.g., the post-graduate entrance examination).

In the control condition, three short videos presented for participants were all about natural scenery. For college students, it is indeed less common to view natural scenery documentary on social media platforms. The underlying logic was that, in the control condition, we sought to create a situation without priming the mindset of social media use. In a pretest (*n* = 32), we asked participants to report perceived quality and visual attractiveness of videos across the two conditions. They declared that they did not perceive significant differences for the selected videos, *t*s(31) < 1.23, *p*s > 0.23. We also asked participants to report their frequencies of accessing such types of videos on social media platforms. The results showed that the mean values of accessing the three activities in the experimental condition were significantly higher than the median 4 on the 7-point scale (*M*s = 5.37, 4.81, 4.50, respectively), *t*s(31) > 2.1, *p*s < 0.05, while the mean value of accessing the natural scenery documentary was significantly lower than the median (*M* = 2.94), *t*(31) = −4.33, *p* < 0.001. During the session of the experiment, we sent the video links to participants’ cellphone and they watched such videos on their own cellphone. Upon completing the task of watching short videos, they were asked to put their cellphones away and continue the following task.

#### 5.2.2. The measure of upward social comparison

Following Vogel et al. (2014), we measured participants’ upward social comparison by explicitly asking “when comparing yourself to others on social media, to what extent do you focus on people who are better off than you?” Participants needed to give their answer on the 5-point scale (1 = *never*, 5 = *always*), with a higher value indicating higher upward social comparison.

#### 5.2.3. The measure of self-esteem

As did in Study 1, the Chinese version of the Rosenberg Self-Esteem Scale was used to measure participants’ self-esteem. According to the recommendation by Alfasi (2019), we instructed participants to report their feeling “at this moment” when they filled out the scale. The internal inconsistency coefficient of the scale was 0.87.

#### 5.2.4. The measure of social anxiety

Following previous research (Carruthers et al., 2019), we asked participants to report to what extent they felt the listed 10 emotions at this moment on the 11-point scale (0 = *not at all*, 10 = *very much*). The 10 emotions included happy, excited, anxiety, proud, fearful, nervous, scared, guilty, grateful, and enthusiastic, but only the score on the anxiety item was analyzed (Carruthers et al., 2019). A higher value represents higher social anxiety.

#### 5.2.5. The measure of demographic information

As did in Study 1, we asked participants to report their gender and age after completing the above several scales. Because residential location did not significantly correlate with any variables in Study 1, we did not collect this information in Study 2. Additionally, considering that subjective social status in nature represented participants’ subjective evaluations toward themselves and may be susceptible to the experimental manipulation, we thus did not collect this information either.

### 5.3. Procedure

Study 2 was conducted in the lab. At the appointed time, participants arrived at the lab in a group of 6~8 people. Upon arrival, we introduced the academic purpose of the study for participants, and they needed to assign the informed consent prior to the formal task. After that, we sent the video links to participants and instructed them to view these short videos on their own cellphone. Then, participants successively reported their upward social comparison, perceived self-esteem, perceived anxiety, and demographic information. When they completed all tasks, we gave each participant 10 RMB as a return for their participation. We explained any questions raised by participants in a detailed way. The whole experiment lasted for about 25~30 min.

### 5.4. Results

#### 5.4.1. Descriptive results in each condition

Means, standard deviations, and correlations among variables were provided in Table 3. As shown in Table 3, there were significantly negative correlations between upward social comparison and self-esteem in both conditions, *r*s = −0.27, −0.54, respectively, *p*s < 0.05. In contrast, there were significantly positive correlations between upward social comparison and social anxiety in both conditions, *r*s = 0.38, 0.23, respectively, *p*s < 0.05. Additionally, there were significantly negative correlations between self-esteem and social anxiety in both conditions, *r*s = −0.37, −0.32, respectively, *p*s < 0.01. On the whole, Study 2 showed the same descriptive-results pattern with Study 1.

**Table 3:** Means, standard deviations, and correlations among variables in each condition of Study 2.

	*M*	*SD*	1	2	3	4
		Experimental condition				
Upward social comparison	4.16	0.81	1			
Self-esteem	26.31	4.55	−0.27^*^	1		
Social anxiety	3.73	1.57	0.38^**^	−0.37^**^	1	
Age	19.48	0.40	0.01	−0.02	−0.08	1
		Control condition				
Upward social comparison	3.79	0.93	1			
Self-esteem	28.47	4.56	−0.54^**^	1		
Social anxiety	2.25	1.45	0.23^*^	−0.32^**^	1	
Age	20.07	1.13	−0.07	−0.14	−0.02	1

M, mean; SD, standard deviation. ^*^p < 0.05, ^**^p < 0.01.

#### 5.4.2. The comparisons for the key variables between two conditions

We conducted several independent-samples *t-*tests to examine whether there were any differences for upward social comparison, self-esteem, and social anxiety between the experimental and control conditions. The results showed that participants in the experimental condition reported that they made more upward social comparisons when using social media than those participants in the control condition (*M*s = 4.16, 3.79, respectively), *t*(173) = 2.77, *p* = 0.006, *d* = 0.42. The independent-samples *t-*test for self-esteem showed that participants in the experimental condition reported lower self-esteem than participants in the control condition (*M*s = 26.31, 28.47, respectively), *t*(173) = −3.14, *p* = 0.002, *d* = 0.47. And the independent-samples *t-*test for social anxiety showed that participants in the experimental condition reported higher social anxiety than participants in the control condition (*M*s = 3.73, 2.25, respectively), *t*(173) = 6.46, *p* < 0.001, *d* = 0.98.

#### 5.4.3. The mediating roles of upward social comparison and self-esteem

To test the possibly mediating roles of upward social comparison and self-esteem between social media exposure condition and social anxiety in Study 2, we coded social media exposure condition as a dummy variable (0 = control condition, 1 = experimental condition) and then applied the Macro PROCESS (model 6) by Hayes (2013) to examine the mediating mechanisms between social media use and social anxiety. The results showed that the overall model was significant, *R*^2^ = 0.09, *F*(3, 171) = 5.35, *p* = 0.0015. As shown in Table 4, the direct effect between social media use and social anxiety was significant, *f* = 0.67, 95% CI [0.39, 0.94], suggesting the same result-pattern with Study 1. Additionally, Table 4 showed that the single-mediating role of upward social comparison, and the chain-mediating role of upward social comparison and self-esteem were small but both significant, *f*s = 0.06, 0.04, respectively, 95% CI [0.01, 0.14], [0.01, 0.09], respectively, thus keeping in line with Study 1. In Study 2, an unexpected finding was that the mediating role of self-esteem between social media use and social anxiety was significant, *f* = 0.09, 95% CI [0.03, 0.21].

**Table 4:** The direct and indirect effects between social media exposure condition and social anxiety in Study 2.

	Effect size	Boot SE	95%LLCI	95%ULCI	Ratio
Direct effect					
Social media exposure → social anxiety	0.67	0.14	0.39	0.94	77.90%
Indirect effect					
Social media exposure → upward social comparison → social anxiety	0.06	0.03	0.01	0.14	6.98%
Social media exposure → upward social comparison → self-esteem → social anxiety	0.04	0.02	0.01	0.09	4.65%
Social media exposure → self-esteem → social anxiety	0.09	0.04	0.03	0.21	10.47%

SE, standard error; LLCI, lower limit of confidence interval; ULCI, upper limit of confidence interval.

**Figure 2:**
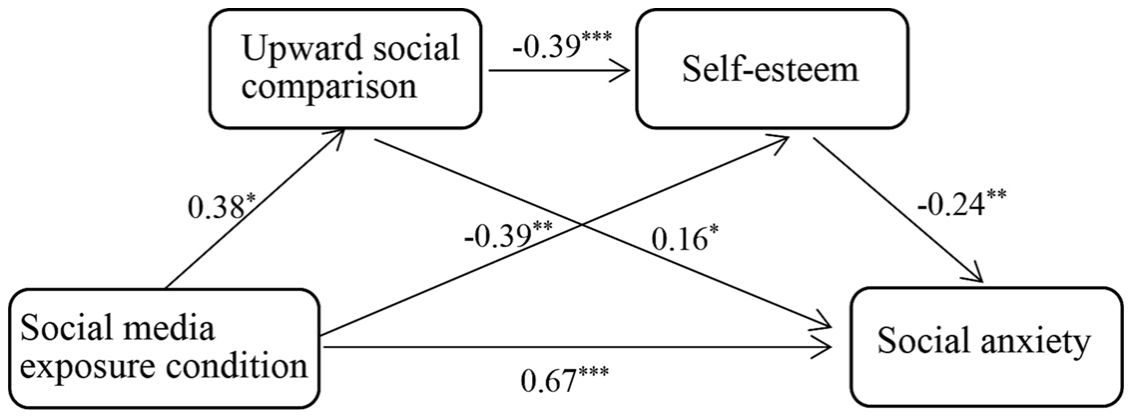
The mediating mechanisms between social media exposure condition and social anxiety in Study 2 were illustrated schematically. ^*^p < 0.05, ^**^p < 0.01, ^***^p < 0.001.

Specific coefficients were provided in Figure 2. As displayed in Figure 2, social media exposure condition had a significantly positive prediction on upward social comparison, *β* = 0.38, *p* = 0.02, and upward social comparison further had a significantly positive prediction on social anxiety, *β* = 0.16, *p* = 0.03, suggesting the mediating role of upward social comparison. Besides producing a significant prediction on social anxiety, upward social comparison also had a significantly negative prediction on self-esteem, *β* = −0.39, *p* < 0.001, and self-esteem in turn significantly and negatively predicted social anxiety, *β* = −0.24, *p* = 0.001, demonstrating the chain-mediating role of upward social comparison and self-esteem. In addition, social media exposure condition was found to have a significantly negative prediction on self-esteem, *β* = −0.39, *p* = 0.008, and self-esteem also had a significantly negative prediction on social anxiety, *β* = −0.24, *p* = 0.001, supporting the mediating role of self-esteem between social media exposure condition and social anxiety.

### 5.5. Discussion

In Study 2, we conducted a lab experiment in which we assigned participants to the experimental and control conditions, and then compared participants’ social anxiety between the two conditions. In the experimental condition, participants were exposed to several wonderful and excited experiences shared by others, which actually also belonged to the content that they often view on social media platforms in daily life. In the control condition, participants were exposed to several short videos related to natural scenery. The results showed that participants viewing wonderful and excited experiences of others (the experimental condition) reported higher social anxiety than participants viewing natural scenery documentaries (the control condition), which suggested that social media use in daily life may increase users’ social anxiety. By demonstrating the causality between social media exposure and social anxiety, Study 2 thus provided compelling evidence for Hypothesis 1.

When social media exposure condition was coded as a dummy variable, we investigated the mediating roles of upward social comparison and self-esteem between social media exposure condition and social anxiety. The results revealed the single-mediating role of upward social comparison and the chain-mediating role of upward social comparison and self-esteem between social media exposure condition and social anxiety, thus providing collaborating evidence for our Hypothesis 2 and 3. Additionally, Study 2 also revealed an unexpected finding that self-esteem played a mediating role between social media exposure and social anxiety. We attempted to explain this unexpected finding in terms of the self-evaluation maintenance model (SEM) proposed by Tesser and Campbell (1980) and Tesser et al. (1988), which contends that an individual’s self-esteem will be threatened when confronting other in-group members who are superior than the individual on ability domains. For example, previous research has found that college students may perceive lower self-esteem when they were exposed to other excellent college students (Blanton et al., 2000). In the experimental condition of Study 2, one of the presented short-videos documented a college graduate sharing the experiences that how she successfully passed the post-graduate entrance examination and entranced Tsinghua University, a top university that many Chinese college students have been longing for. Obviously, the protagonist is an excellent college student and this may yield a threaten for participants’ self-esteem. As a consequence, participants in the experimental condition perceived lower self-esteem, and lower self-esteem further contributed to higher social anxiety. Considering this unexpected mediating role of self-esteem was not our major concern, we thus did not make further investigation on this issue. We will keep an eye on this issue in future research.

There was another finding that may need our explanation. That is, considering that participants in the control condition just viewed natural scenery documentaries, we asked participants in both conditions to report the tendency of upward social comparison when using social media rather than viewing short videos. Even so, participants in the experimental condition still report significantly stronger upward social comparison than participants in the control condition. This difference, in nature, may reflected a priming effect, a widespread phenomenon in daily life (for a detailed discussion, see Higgins, 2012). Past research has suggested that when individuals are exposed a specific kind of stimuli, the knowledge accessibility relevant to the stimuli will be elevated and such knowledge constructs correspondingly get priority in the following information processing (Higgins et al., 1982; Higgins and Brendl, 1995; Bargh et al., 1996). For instance, the research regarding stereotypes demonstrates that individuals may form impressions or make decisions following the guidance of stereotypes when stereotypic knowledge constructs are accessible, but if counter-stereotypic knowledge constructs are accessible, individuals may also rely on counter-stereotypic information to process information (Power et al., 1996; Finnegan et al., 2015). With respect to the current research, participants in the experimental condition were presented with the content that college students often browse on social media platforms, and such videos were more likely to activate knowledge constructs relevant to social media use than natural scenery documentaries. As a result, those experiences suffering from upward social comparison may also be activated (Steers et al., 2014; Liu et al., 2017). From this perspective, it seemed to be reasonable and acceptable for the finding that participants in the experimental condition reported more upward social comparisons than participants in the control condition.

## 6. General discussion

In the represent research, we conducted two studies to investigate the effect of social media use on social anxiety and the mechanisms underlying the effect. The results showed that higher social media use intensity led to higher social anxiety. Besides the direct effect, social media use also increased social anxiety of social media users via the single-mediating role of upward social comparison and the chain-mediating role of upward social comparison and self-esteem. The research carried some implications in the field of social media use and social anxiety.

### 6.1. The effect of social media use on social anxiety

As we have discussed in the introduction section, although past research has consistently demonstrated the significant correlation between social media use and social anxiety, the causal evidence that higher social media user causes higher social anxiety is still scarce (Davidson and Farquhar, 2014; Xie and Karan, 2019; Jiang and Ngien, 2020; Sternberg et al., 2020). The current research extended prior research in the field of social media use and social anxiety by providing the first evidence that excessive social media use could increase social anxiety. Moreover, in Study 2, participants were temporarily exposed to the content that they often view on social media platforms or the natural scenery documentaries that they are less likely to view on social media platforms. The results showed that, even this temporary exposure in the artificial laboratory-setting, still induced higher social anxiety of participants, which significantly strengthened our confidence to conclude that social media use will increase social anxiety. Interestingly, it has been widely documented that individuals with social anxiety are inclined to avoid the face-to-face interaction and construct their social networks on social media platforms, so that they can alleviate their social anxiety (Shepherd and Edelmann, 2005; Caplan, 2007; Carruthers et al., 2019). However, according to our results, social anxious individuals may experience less social anxiety in a specific online interaction, but their social anxiety may be further exacerbated in the long run. In other words, the online interaction indeed cannot replace the face-to-face interaction.

We noticed that when the indirect effects were taken into account, the direct effect of social media use on social anxiety was still significant across two studies, which was inconsistent with previous research by Jiang and Ngien (2020). We speculated that this inconsistency may be because the participants included in the research were all college students. Specifically, compared to other age groups, college students often have more free time and also spend more time on various of social media platforms (She et al., 2023). And correspondingly, prior research suggests that social anxiety actually is quite common among college students and the majority of them have experienced social anxiety in social situations from time to time (Purdon et al., 2001). For individuals with social anxiety, when encountering some cues relevant to social interactions, they may spontaneously imagine an anxiety-provoking social situation and look at themselves from an observer’s perspective, which will further increase their social anxiety (Hackmann et al., 2000; Hirsch et al., 2004; Morrison and Heimberg, 2013). In the current research, maybe our explicit declaration that participants would complete the survey relevant to social media use and social anxiety immediately induced college students’ social anxiety in both studies, resulting in relatively strong direct effect of social media use on social anxiety. Of course, we must realize that this explanation is tentative and premature, and needs further investigation in future work.

### 6.2. The mediating roles of upward social comparison and self-esteem

Consistent with previous research, the present research found that individuals with higher social media use intensity experienced more upward social comparisons, and upward social comparisons further increased social anxiety (Jiang and Ngien, 2020). In simple words, social media use would increase users’ social anxiety via the mediating role of upward social comparison. It should be pointed out, although prior research has revealed a series of negative effects on mental health, several studies have revealed some factors that may alleviate the negative effects of upward social comparison on mental health (Cramer et al., 2016; Kong et al., 2021; Latif et al., 2021). For example, prior research indicates that if individuals realize that they are making social comparison with superior others, they may hold positive evaluations for themselves and tend to believe they can do as well as the reference target (Collins, 2000; Suls et al., 2002). Following this logic, in daily life, we may can relieve social anxiety of social media users by explicitly reminding them that they are suffering from upward social comparison when using social media. Besides, we also can alleviate social anxiety of social media users by encouraging them to use social media with a self-improvement motive, because past research has suggested that, for individuals with a self-improvement motive, even exposed to superior others, they are still inclined to believe that they can improve the ability of themselves (Lockwood and Kunda, 1997; Cramer et al., 2016).

Beside the single-mediating role of upward social comparison, we also found that social media use could increase social anxiety through the chain-mediating role of upward social comparison and self-esteem. This mediation path inspired us that we may can alleviate social anxiety of social media users by elevating their self-esteem. For instance, past research found that positive feedback received from social media platforms enhanced users’ self-esteem while negative feedback received from social media platforms decreased users’ self-esteem (Valkenburg et al., 2006). And compared to casual acquaintances, individuals are more likely to receive feedback from close friends (Carr et al., 2016). Thus, for individuals with social anxiety, it is better for them to construct a “small but intimate” online social network than a “large but loose” online social network. Because the former enables users to receive more positive feedback from close friends, which will be helpful for alleviating social anxiety. Moreover, close friends are considered to be a primary source of social support and social support is an important protective-factor for self-esteem (Goodwin et al., 2004; Rozzell et al., 2014). On a broader level, considering that social media use may increase users’ social anxiety through decreasing self-esteem, those measures that are helpful for elevating self-esteem may deserve an attempt to examine whether they also can relieve social anxiety of social media users.

### 6.3. Limitations and future work

There were several limitations existing in the current research. Firstly, the indirect effect was relatively small in comparison with the direct effect across two studies. As we have explained in the previous section, this may be because some cues relevant to social interactions immediately induced social anxiety of college students (e.g., the instruction that we explicitly told participants that they would take part in a survey about social media use and social anxiety), resulting in the relatively small indirect effect across two studies. Despite this, considering that previous research has revealed similar results-pattern with the current research (Jiang and Ngien, 2020), we still have confidence for our findings and their implications for deepening our understanding about how social media use will increase social anxiety of social media users.

Secondly, in Study 2, we revealed an unexpected mediating-effect that social media use produced an effect on social anxiety via the mediating role of self-esteem. According to our explanation, this unexpected effect may result from presenting an introduction about an excellent undergraduate who had successfully entered Tsinghua University. That means, viewing different kinds of content on social media might have different psychological consequences for social media users. Additionally, the event that an individual successfully entered Tsinghua University may occasionally raise social status concerns of participants, which in turn increased social anxiety of participants. Given that, in future work, we can attempt to explore whether the effect of social media use on social anxiety will vary with the presented content on social media.

Thirdly, excessive social media use has been found to have close connections with a series of negative mental consequences. However, to date, there is little causal evidence that social media use will increase social anxiety. So, the primary goal of the present research was to provide causal evidence for whether and how social media use will increase social anxiety. Nevertheless, we must realize that prior research has shown that individuals with social anxiety are more likely to use social media to compensate for their social deficit (Lee-Won et al., 2015; Stănculescu and Griffiths, 2022), displaying the reversed pattern with the present research. For this phenomenon, we speculate that the relationship between social media use and social anxiety may not be a simple causality in a single direction. Rather, there may be a mutually reinforcing relationship between social media use and social anxiety—individuals with social anxiety tend to excessively use social media, which will further exacerbate their social anxiety. Considering this possibly mutually reinforcing relationship between social media use and social anxiety, it may be necessary to develop a unified theoretical framework to interpret this relationship.

Fourthly, in Study 2, the baseline social anxiety of participants was not taken into accounted, which may constitute a confounding factor for our findings. Because prior literature has indicated that individuals with high social anxiety are more vulnerable to risk factors in external environment than those with low social anxiety (e.g., Mitchell and Schmidt, 2014). On a broad level, to thoroughly answer the question whether social media use will elevate social anxiety, we need to take possible individual differences into account in future research (e.g., lower trait self-esteem individuals are more likely to suffer from social anxiety than higher trait self-esteem individuals; Fatima et al., 2017). Given such potential limitations, in future research, we need to replicate our findings in both community and clinical samples, and compare potential results-pattern differences between the two samples.

Finally, due to the regulation of Chinese government, many social media platforms popular in Western society fail to provide service in China-Mainland. Correspondingly, those social media platforms popular in China-Mainland actually have a relatively small user base. Given that, although the current research showed the similar results-pattern with previous research in Western society (Davidson and Farquhar, 2014; Xie and Karan, 2019; Sternberg et al., 2020), it may be still necessary for us to examine to what extent our findings can be generalized to Western society.

## 7. Conclusion

By conducting a questionnaire survey (Study 1) and a lab experiment (Study 2), the current research investigated the effect of social media use on social anxiety of social media users, and the underlying mechanisms. The results demonstrated that higher social media user intensity would induce higher social anxiety of social media users. In addition to the direct effect, higher social media use would lead to higher upward social comparison and higher upward social comparison would increase social anxiety. Besides, upward social comparison induced by social media use may also have a detrimental effect self-esteem, which further contributed to increasing social anxiety. The current research deepens our understanding of how social media use will increase social anxiety and also carries implications for how we can alleviate social anxiety induced by excessive social media use.

## Data availability statement

The raw data supporting the conclusions of this article will be made available by the authors, without undue reservation.

## Ethics statement

The studies involving humans were approved by the Ethics Committee of Taishan University. The studies were conducted in accordance with the local legislation and institutional requirements. The participants provided their written informed consent to participate in this study.

## Author contributions

FY provided the research design and wrote the manuscript. ML collected the data, involved in the research, and conducted data analyses. YH corroborated with FY to conduct data analyses. All authors contributed to the article and approved the submitted version.

## Funding

This research was supported by the project of Social Sciences of Shandong Province (no. 22DJYJ15).

## Conflict of interest

The authors declare that the research was conducted in the absence of any commercial or financial relationships that could be construed as a potential conflict of interest.

## Publisher’s note

All claims expressed in this article are solely those of the authors and do not necessarily represent those of their affiliated organizations, or those of the publisher, the editors and the reviewers. Any product that may be evaluated in this article, or claim that may be made by its manufacturer, is not guaranteed or endorsed by the publisher.

## Footnotes

^1^ https://weixin.qq.com/

^2^ https://www.bilibili.com/
